# Improvements in mood symptoms, cognitive symptoms and functioning in outpatients with mdd in greece treated with vortioxetine: A patient-rated evaluation

**DOI:** 10.1192/j.eurpsy.2021.895

**Published:** 2021-08-13

**Authors:** A. Galanopoulos, E. Tsiolka, P. Ntounas, D. Mpelimpasakis, D. Karakoutas, N. Sidiropoulos, I. Mpougiouklis, K. Kyziridis, E. Papalexi

**Affiliations:** 1 Medical, Lundbeck Hellas, Athens, Greece; 2 Psychiatric, Private Office, Athens, Greece; 3 Psychiatric, Private Office, Thesaloniki, Greece; 4 Medical, Lundbeck Hellas, ATHENS, Greece

**Keywords:** Mood, Functionality, Patient-rated, Vortioxetine

## Abstract

**Introduction:**

Functional recovery is the contemporary treatment goal in Major Depressive Disorder (MDD). Although consistency among physician and patient expectations may influence the therapeutic result (Demyttenaere K et al, 2011), patients’ perceptions are not always fully captured. Vortioxetine,a multimodal antidepressant, has shown encouraging data in achieving functional recovery, improving both mood and cognitive symptoms (Mahableshwarkar AR et al, 2015).

**Objectives:**

The aim of the study was to assess the effectiveness of vortioxetine on mood symptoms, cognitive symptoms and functionality, assessed by patient-rated tools, in MDD outpatients in Greece.

**Methods:**

In this non-interventional study, vortioxetine was administered as flexible dosing (5-20 mg/d). Mood symptoms, cognitive symptoms and functioning were assessed by the patient-rated scales PHQ-9, PDQ-D and SDS respectively, at baseline, 1 and 3 months. Repeated measures analysis of variance and t-test were used for the statistical analyses.

**Results:**

336 patients participated in the study. PHQ-9 score ±SD decreased from 16.1±5.3, to 10.0±5.7 and 4.6±4.5, PDQ-D score ±SD decreased from 37.3±16.6 to 23.1±14.8 and 12.0±10.6, SDS Score ±SD decreased from 18.7±5.3 to 12.9±5.9 and to 7.8±6.5, at baseline, 1 and 3 months, respectively. The 3 SDS subscales: work/school life improved from 5.8±2.4 to 4.2±2.2 and 2.6±2.2, social life improved from 6.6±2.0 to 4.5±2.2 and 2.7±2.3 and family life improved from 6.3±2.0 to 4.3±2.1 and 2.6±2.3 -baseline, 1 and 3 months, respectively (p<0.001 for all paired comparisons).

**Conclusions:**

MDD patients in Greece treated with vortioxetine significantly improved on mood symptoms, cognitive symptoms and functioning, enriching the already published efficacy data which is mostly based on clinician-rated scales.

**Conflict of interest:**

A. Galanopoulos and E. Papalexi are full-time employees in Lundbeck Hellas.

